# A meta-analysis of *ABCG2* gene polymorphism and non-small cell lung cancer outcomes

**DOI:** 10.1590/1678-4685-GMB-2018-0234

**Published:** 2020-02-14

**Authors:** Lei Fu, Rong Wang, Ling Yin, Xiaopu Shang, Runtong Zhang, Pengjun Zhang

**Affiliations:** ^1^ Core Laboratory of Translational Medicine Core Laboratory of Translational Medicine Chinese PLA General Hospital Beijing China Core Laboratory of Translational Medicine, Chinese PLA General Hospital, Beijing, China.; ^2^ National Defence University of PLA National Defence University of PLA Joint Logistics College Beijing China Joint Logistics College, National Defence University of PLA, Beijing, China; ^3^ National Research Institute for Family Planning National Research Institute for Family Planning Beijing China National Research Institute for Family Planning, Beijing, China.; ^4^ Beijing Jiaotong University Beijing Jiaotong University School of Economics and Management Beijing China Beijing Jiaotong University, Beijing, School of Economics and Management, China.; ^5^ Peking University Cancer Hospital and Institute Peking University Cancer Hospital and Institute Department of Interventional Therapy Beijing China Peking University Cancer Hospital and Institute, Department of Interventional Therapy, Key Laboratory of Carcinogenesis and Translational Research (Ministry of Education of China), Beijing, China.

**Keywords:** ABCG2, polymorphism, Non-small cell lung cancer, chemotherapy, meta-analysis

## Abstract

We aimed to analyze the correlation between *ABCG2* gene polymorphisms of 34 GG/(GA + AA) loci, 421 CC/(AC + AA) loci, and non-small cell lung cancer (NSCLC) therapeutic effects via meta-analysis. With key words, the databases PubMed and EMBASE were searched for clinical studies on ABCG2 polymorphism and NSCLC. *RR* and 95% *CI*s were used to compute combined effects, followed by heterogeneity testing. Publication bias was examined using the funnel plot method. Review Manager 5.3 software was used for the meta-analysis. Ten studies were included. No evidence of heterogeneity exists in these studies. The results indicate that two polymorphic loci of *ABCG2* gene (34 G>A, and 421 C>A) had no relationship with the curative effect of chemotherapy for NSCLC, except ABCG2 34G>A, which had a significant relationship with the skin toxicity complication. There was no significant relationship between these polymorphisms and complications (skin toxicity, diarrhea, interstitial pneumonia, liver dysfunction, and neutropenia). Begg’s test and Egger’s test indicated that there was no obvious publication bias. The meta-analysis indicated that there was no significant correlation between *ABCG2* gene polymorphism and NSCLC outcomes.

## Introduction

Adenosine triphosphate-binding cassette sub-family G member 2 (ABCG2) performs certain physiological functions *in vivo*, such as maintaining cell homeostasis (Susanto *et al.*, 2008), the blood-brain barrier (Cisternino *et al.*, 2004; Eisenblätter *et al.*, 2003), disease susceptibility (Phippsgreen *et al.*, 2010), and pharmacokinetics (Lee *et al.*, 2015). Additionally, studies have reported that it has an effect on multi-drug resistance of chemotherapeutic agents, such as mitoxantrone and camptothecin analogues (Yoshikawa *et al.*, 2004, Nakagawa *et al.*, 2006). Previous studies have suggested that several naturally occurring single-nucleotide polymorphisms (SNPs, variations in a single nucleotide at a specific position in the genome), in the ABCG2 gene may affect the expression and function of ABCG2 protein (Kobayashi *et al.*, 2005; Lepper *et al.*, 2005). More than 80 SNPs have been identified in the *ABCG2* gene (Sharom 2008). Specifically, ABCG2 polymorphism caused by the 421 locus change in the fifth exon could lead to a decrease in ABCG2 protein expression, which in turn affects the removal and absorption of pravastatin (Oh *et al.*, 2013) and simvastatin (Zhou *et al.*, 2013). Chen et al. (2012) have indicated that the ABCG2 421C>A (rs2231142) polymorphism, resulting in a Glu141Lys substitution, is a protective factor for developing cancer. Additionally, ABCG2 34G>A (rs2231137), resulting in a Val12Met substitution, is also well studied and is related to the adverse effect of many drugs that are transported by ABCG2 (Imai *et al.*, 2002).

Lung cancer is the leading cause of cancer-related deaths worldwide, and approximately 85% of lung cancers are non-small cell lung cancer (NSCLC) (Aggarwal *et al.*, 2010). Chemotherapy is a common choice for NSCLC treatment (Reynolds 1995; Ren *et al.*, 2011), while chemoresistance is a challenge during the treatment (Chang 2011). As mentioned above, SNPs in ABCG2 can affect the expression of ABCG2 protein. ABCG2 protein expression is reported to be related to the response of advanced NSCLC patients treated with chemotherapy (Ota *et al.*, 2009). Some studies have focused on investigating the relationships between *ABCG2* gene polymorphism and treatment effects of chemotherapy on NSCLC patients, however no consensus has been reached (Cusatis *et al.*, 2006; Han J Y *et al.*, 2007; Akasaka *et al.*, 2010; Müller *et al.*, 2010; Lemos C *et al.*, 2011; Campa *et al.*, 2012; Mariko *et al.*, 2012; Fukudo *et al.*, 2013; Kobayashi *et al.*, 2015; Chen *et al.*, 2015). Tamura *et al.* (2012) suggest that ABCG2 34G>A would be useful in predicting a worsening of skin rash. Lemos *et al.* (2011) did not find any significant association between the evaluated ABCG2 polymorphisms and response, clinical benefit, time to progression (TTP), or overall survival (OS). Moreover, due to the small sample sizes of the individual studies, there is a need to perform a meta-analysis to combine them and systematically analyze the relationships between *ABCG2* gene polymorphism and treatment effects among NSCLC patients.[Bibr B28]

Therefore, this study aims to explore the prognosis value of *ABCG2* gene polymorphism on the chemotherapy effect of NSCLC through a systematic review of studies and meta-analysis.

## Material and Methods

### Data sources

The search strategy was pre-designed. The databases PubMed and EMBASE were searched for studies on *ABCG2* gene polymorphism and NSCLC outcomes published before December 3, 2018. The keywords included: [‘non-small cell lung cancer’ OR ‘NSCLC’ OR ‘squamous cell lung cancer’ OR ‘lung adenocarcinoma’ OR ‘large cell lung cancer’] AND [‘ATP-binding cassette sub-family G member 2’ OR ‘ABCG2’ OR ‘breast cancer resistance protein’ OR ‘BCRP’ OR ‘CDw338’ OR ‘mitoxantrone resistance protein’ OR ‘MRP’ OR ‘ABCP’] AND [‘polymorphism’ OR ‘polymorphisms’ OR ‘genetic’ OR ‘variation’ OR ‘genotyping’ OR ‘SNP’].

### Inclusion criteria and quality assessment

The inclusion criteria were 1) clinical studies with NSCLC patients as cases; 2) studies that investigated the correlation between *ABCG2* gene polymorphisms and NSCLC treatment effects; 3) studies that reported the frequencies of gene types and alleles or from which these data can be calculated; 4) studies that reported curative effect indicators such as progression free survival (PFS), overall survival (OS), mortality risk, and response; and adverse effect indicators such as drug-induced diarrhea, skin toxicity, liver dysfunction, and interstitial pneumonia; 5) reviews, reports, comments or letters were excluded. Newcastle-Ottawa Scale (NOS) (Stang, 2010) was used for quality assessment.

### Data extraction

The following data from the included studies was extracted independently by two researchers, including first author, publication year, distribution of ethnic groups, distribution and frequencies of genotypes and alleles, and the gender and age of patients in each study. If there was inconsistency during data extraction, discussion with a third researcher was initiated until an agreement was reached.

### Statistical analysis

Meta-analysis was conducted using the Review Manager Version 5.3 (2008). Mortality risk was combined using HR and 95% *CI*. *RR* and 95% *CI*s (m (mutation)/m + w (wild)/m vs. w/w) were used to calculate the combined effect sizes of the other indicators. Heterogeneity test was conducted according to the chi-square-based *Q* test (Lau *et al.*, 1997) and *I*^2^ statistic. If there was significant heterogeneity (*P* < 0.05, *I*^2^ > 50%), the random-effect model (by Dersimonian-Laird method) was used to pool the effect sizes; otherwise, the FE model (by Mantel-Haenszel method) was used. Subgroup meta-analysis based on chemotherapeutics, race, and grade of toxicity was performed. Begg’s test and Egger’s test were used to examine publication bias for studies with the largest number of publications included. All tests were two-sided, with a significance threshold of *P* < 0.05.

## Results

### Study selection and the characteristics of correlational studies

**Figure 1:**
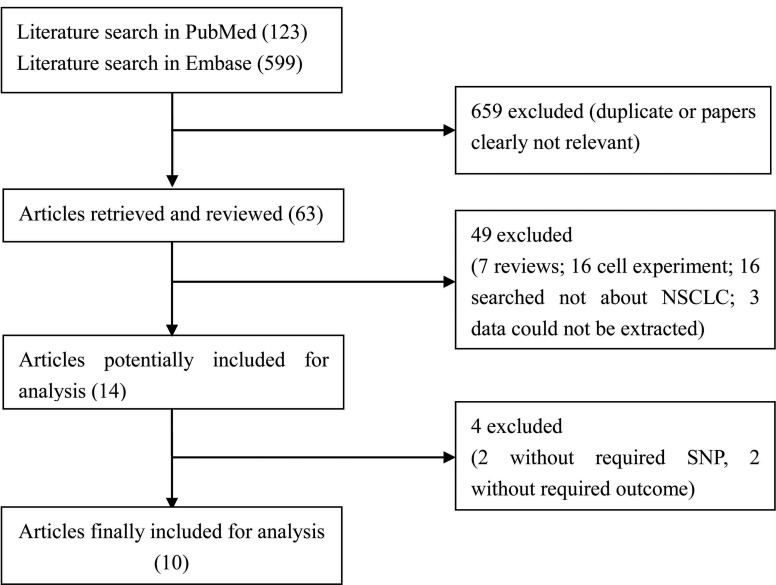
Flow chart of literature search and study selection.

Figure 1 shows the study selection procedure. Firstly, a total of 722 studies (123 in PubMed and 599 in EMBASE) were searched. After removal of duplicates or irrelevant studies, 63 studies remained for reading of the full text and abstract. Of these, 10 reviews and 20 cell experiments, 16 non-NSCLC related studies and three without extractable data were rejected, leaving 14 studies. Additionally, two studies without data associated with 34 G>A and 421 C>A, and two studies without the correlation between ABCG2 polymorphism and efficacy and side effects of chemotherapy were excluded. Finally, a total of 10 studies (Cusatis *et al.*, 2006; Han *et al.*, 2007; Muller *et al.*, 2009; Akasaka *et al.*, 2010; Lemos *et al.*, 2011; Tamura *et al.*, 2012; Fukudo *et al.*, 2013; Chen *et al.*, 2015; Kobayashi *et al.*, 2015; Ma *et al.*, 2017) were included in this meta-analysis.[Bibr B3] [Bibr B4]

The characteristics of correlational studies are listed in Table 1. These studies were mainly conducted in China, Japan, Germany, Italy, and Korea. The patients mainly were at stages III–IV. The chemotherapy regimens included Etoposide + Gemcitabine + Platinum-based drugs, Gefitinib and Erlotinib. The studies had relatively high-quality scores of 5–7 (Table 2).

**Table 1:** Characteristics of the included studies.

Author	Year	Area (race)	No. of patients	Chemotherapy regimen	Stage	Evaluation criteria
Akasaka	2010	Japan (Asian)	75	Gefitinib	I-IV	WHO
Chen	2015	China (Asian)	70	gefitinib, erlotinib and icotinib	I-II	ECOG
Cusatis	2006	Italy (Caucasian)	173	Gefitinib	I-IV	WHO
Fukudo	2013	Japan (Asian)	88	Erlotinib	IIIB-IV	ECOG
Han	2007	Korea (Asian)	107	Irinotecan and cisplatin	IIIB-IV	ECOG
Kobayashi	2014	Japan (Asian)	31	Gefitinib	IIIB-IV	CTCAE
Lemos	2011	Italy (Caucasian)	94	Gefitinib	IIIB-IV	ECOG
Ma	2017	China (Asian)	59	Gefitinib	IIIB-IV	ECOG
Muller	2009	Germany (Caucasian)	187	Etoposide+Gemcitabine+Platinum-based drugs	II-IV	RECIST
Tamura	2012	Japan (Asian)	83	Gefitinib	I-IV	CTCAE

WHO, World Health Organization; ECOG, Eastern Cooperative Oncology Group; CTCAE, Common Terminology Criteria For Adverse Events; RECIST, Response Evaluation Criteria In Solid Tumors.

**Table 2:** Quality assessment of the included studies with Newcastle-Ottawa quality assessment scale.

First author	Representativeness of the exposed cohort	Selection of the unexposed cohort	Ascertainment of exposure	Outcome of interest not present at start of study	Control for important factor or additional factor	Outcome assessment	Follow-up long enough for outcomes to occur	Adequacy of follow-up of cohorts	Total quality scores
Akasaka 2010	#	--	#	#	--	#	#	#	6
Chen 2015	#	--	#	#	#	#	#	#	7
Cusatis 2006	#	--	#	#	--	#	--	#	5
Fukudo 2013	#	--	#	#	#	#	#	#	7
Han 2007	#	--	#	#	--	#	#	#	6
Kobayashi 2015	#	--	#	--	#	#	#	#	6
Lemos 2011	#	--	#	#	#	#	#	#	7
Ma 2017	#	--	#	#	--	#	#	#	6
Muller 2009	#	--	#	#	#	#	#	#	7
Tamura 2012	#	--	#	#	--	#	#	#	6

### Correlation between *ABCG2* gene polymorphism and treatment effect of NSCLC

The correlations between the polymorphisms of two loci of the *ABCG2* gene and the prognosis of chemotherapy for NSCLC were investigated. The results are displayed in Figures S1      -S8. For the indicators of OS, PFS, mortality (Figures S1 -S3), and interstitial pneumonia (Figure S8), the included literature only report the data related to ABCG2 421C>A. There were no heterogeneities among studies for all curative effect indicators and adverse effect indicators (*P* > 0.05, *I*^*2*^ > 50%), thus the FE model was adopted to combine all effect sizes. The meta-analysis results show that the polymorphism ABCG2 421C>A had no relationship with outcomes of chemotherapy for NSCLC (*P* > 0.05), and ABCG2 34G>A was significantly correlated with skin toxicity (*P* < 0.05) (Figures S1      -S8).

### Subgroup analysis

Subgroup analysis of skin toxicity and diarrhea of 421 loci CC/(AC + AA) based on the chemotherapeutics (gefitinib *vs.* others), races (Asian *vs.* Caucasian) and grade of toxicity (Grade f 1 vs. 0, Grade y 2 vs. Grade < 2) were performed. The results show that ABCG2 421C>A had no influence on skin toxicity or diarrhea (*P* > 0.05, Table 3).

**Table 3:** Subgroup analyses of ABCG2 421C>A (AA+CA vs. CC).

Subgroups	Adverse events		*P*_value_	Test for heterogeneity	
	N	OR(95%CI)		I^2^ (%)	*P*
**Association between ABCG2 421C**>A **and skin toxicity**					
**Race**					
Asian	5	0.88 (0.74, 1.05)	0.17	0%	0.81
Caucasian	2	0.99 (0.78, 1.25)	0.95	0%	0.54
**Chemotherapy regimen**					
Gefitinib	6	0.89 (0.77, 1.04)	0.16	0%	0.71
Others	1	1.00 (0.68, 1.48)	0.98	--	--
**Grade of toxicity**					
Grade ≥	5	0.91 (0.78, 1.06)	0.23	0%	0.64
Grade(0	2	0.92 (0.65, 1.30)	0.63	0%	0.49
**Association between ABCG2 421C**>A **and diarrhea**					
**Race**					
Asian	6	0.99 (0.70, 1.38)	0.93	22%	0.27
Caucasian	2	0.82 (0.60, 1.12)	0.22	0%	0.43
**Chemotherapy regimen**					
Gefitinib	6	0.80 (0.62, 1.02)	0.07	0%	0.97
Others	2	1.69 (0.86, 3.33)	0.13	48%	0.16
**Grade of toxicity**					
Gradeto	5	0.81 (0.63, 1.04)	0.10	0%	0.93
Grade63	3	1.44 (0.76, 2.71)	0.26	43%	0.17

### Publication bias

The publication bias test was conducted on “drug-induced diarrhea” of ABCG2 421C>A that had the most included papers. Both Begg’s test and Egger’s test indicated that no publication bias exists (Begg’s test: *P* = 0.386, Egger’s test: *P* = 0.834).

## Discussion

This meta-analysis systematically reviewed *ABCG2* gene polymorphisms and the efficacy and safety of NSCLC treatment. Polymorphisms at two loci of the *ABCG2* gene (34 G>A and 421 C>A) were evaluated. In addition, the qualities of the included studies are relatively high. There is no significant heterogeneity among studies for the entire analysis. Moreover, no publication bias is noted. Furthermore, compared with a recent meta-analysis of Tang *et al.* (2018), which determined whether *ABCG2* gene polymorphisms are associated with the risk of gefitinib-induced toxicity in NSCLC patients, our study added meta-analysis of survival outcomes.

Overall, this meta-analysis did not find a significant relationship between evaluated *ABCG2* gene polymorphisms and the curative effects and adverse effects of chemotherapy of NSCLC, except that ABCG2 34G>A showed a negative relationship with skin toxicity in patients after chemotherapy. However there was only one study (Mariko *et al.*, 2012) on 34G>A, which might have resulted in insufficient power. More studies on 34G>A should be performed.

ABCG2 may have an effect on the multi-drug resistance of chemotherapeutic agents such as mitoxantrone and camptothecin analogues (Nakagawa *et al.*, 2006; Yoshikawa *et al.*, 2004). However, for the NSCLC patients, cisplatin (59.73%) and carboplatin (30.20%) are mostly used (Ren *et al.*, 2011). In the studies included in this meta-analysis, gefitinib is the most widely used, followed by etoposide. Drug resistance to gefitinib and etoposide was not noted. Subgroup analysis based on different chemotherapeutics was performed. There was no significant relationship between the polymorphisms on 421C>A and skin toxicity or diarrhea after treatment for gefitinib or other drugs. Similarly, there were no differences between Asians and Caucasians in the relationship.

This meta-analysis did not limit the types of chemotherapy drugs and included as many studies as possible. Additionally, we added the analysis of survival outcomes. Nevertheless, there were shortcomings in this study, due to the small sample size for some indices, and conclusions from the results should therefore be drawn with caution.

In all, it can be concluded that the ABCG2 polymorphism could not be used as a prognosis indicator of chemotherapy for NSCLC. However, due to the limitations in this study, the results should be interpreted cautiously. More studies with large sample sizes, randomized designs, and unified styles of outcomes are necessary.

## Supplementary material

The following online material is available for this article:

Figure S1 - Meta-analysis of *ABCG2* gene polymorphisms and progression free survival after chemotherapy in the NSCLC for 421 CC/(AC+AA).

Figure S2 - Meta-analysis of *ABCG2* gene polymorphisms and overall survival after chemotherapy in the NSCLC for 421 CC/(AC+AA).

Figure S3 - Meta-analysis of *ABCG2* gene polymorphisms and mortality due to chemotherapy in the NSCLC for 421 CC/(AC+AA).

Figure S4 - Meta-analysis of *ABCG2* gene polymorphism and response to chemotherapy in the NSCLC for 34 GG/(GA+AA), and 421 CC/(AC+AA).

Figure S5 - Meta-analysis of *ABCG2* gene polymorphism and diarrhea due to chemotherapy in the NSCLC for 34 GG/(GA+AA), and 421 CC/(AC+AA).

Figure S6 - Meta-analysis of *ABCG2* gene polymorphism and skin toxicity due to chemotherapy in the NSCLC for 34 GG/(GA+AA), and 421 CC/(AC+AA).

Figure S7 - Meta-analysis of *ABCG2* gene polymorphism and liver dysfunction due to chemotherapy in the NSCLC for 34 GG/(GA+AA), and 421 CC/(AC+AA).

Figure S8 - Meta-analysis of *ABCG2* gene polymorphism and interstitial pneumonia due to chemotherapy in the NSCLC for 421 CC/(AC+AA).
