# Supplementary material for: A meta-analysis of ABCG2 gene polymorphism and non-small cell lung cancer outcomes
Source: Genet Mol Biol. 2020 Feb 14;42(4):e20180234. doi: 10.1590/1678-4685-GMB-2018-0234 (PMC7266279; doi:10.1590/1678-4685-GMB-2018-0234)
Supplement: Supplementary file 4 [file 1415-4757-GMB-42-4-e20180234-s4.pdf]

Supplementary Material to "A meta-analysis of *ABCG2* gene polymorphism and non-small cell lung cancer outcomes"

Study or Subgroup	log[Risk Ratio]	SE	Weight	Risk Ratio IV, Random, 95% CI
**1.4.1 ABCG2 421C>A**				
Akasaka 2010	0.0729	0.2707	17.8%	1.08 [0.63, 1.83]
Chen 2015	0.087	0.1731	43.5%	1.09 [0.78, 1.53]
Han 2007	0.2213	0.2127	28.8%	1.25 [0.82, 1.89]
Lemos 2011	-0.7516	0.9796	1.4%	0.47 [0.07, 3.22]
Muller 2009	0.077	0.3929	8.5%	1.08 [0.50, 2.33]
**Subtotal (95% CI)**			**100.0%**	**1.12 [0.89, 1.40]**
Heterogeneity: Tau² = 0.00; Chi² = 1.09, df = 4 (P = 0.90); I² = 0%				
Test for overall effect: Z = 0.97 (P = 0.33)				
**1.4.2 ABCG2 34G>A**				
Chen 2015	0.1382	0.1916	52.9%	1.15 [0.79, 1.67]
Han 2007	0.0689	0.2141	42.3%	1.07 [0.70, 1.63]
Muller 2009	-0.6539	0.6343	4.8%	0.52 [0.15, 1.80]
**Subtotal (95% CI)**			**100.0%**	**1.07 [0.82, 1.41]**
Heterogeneity: Tau² = 0.00; Chi² = 1.43, df = 2 (P = 0.49); I² = 0%				
Test for overall effect: Z = 0.51 (P = 0.61)				

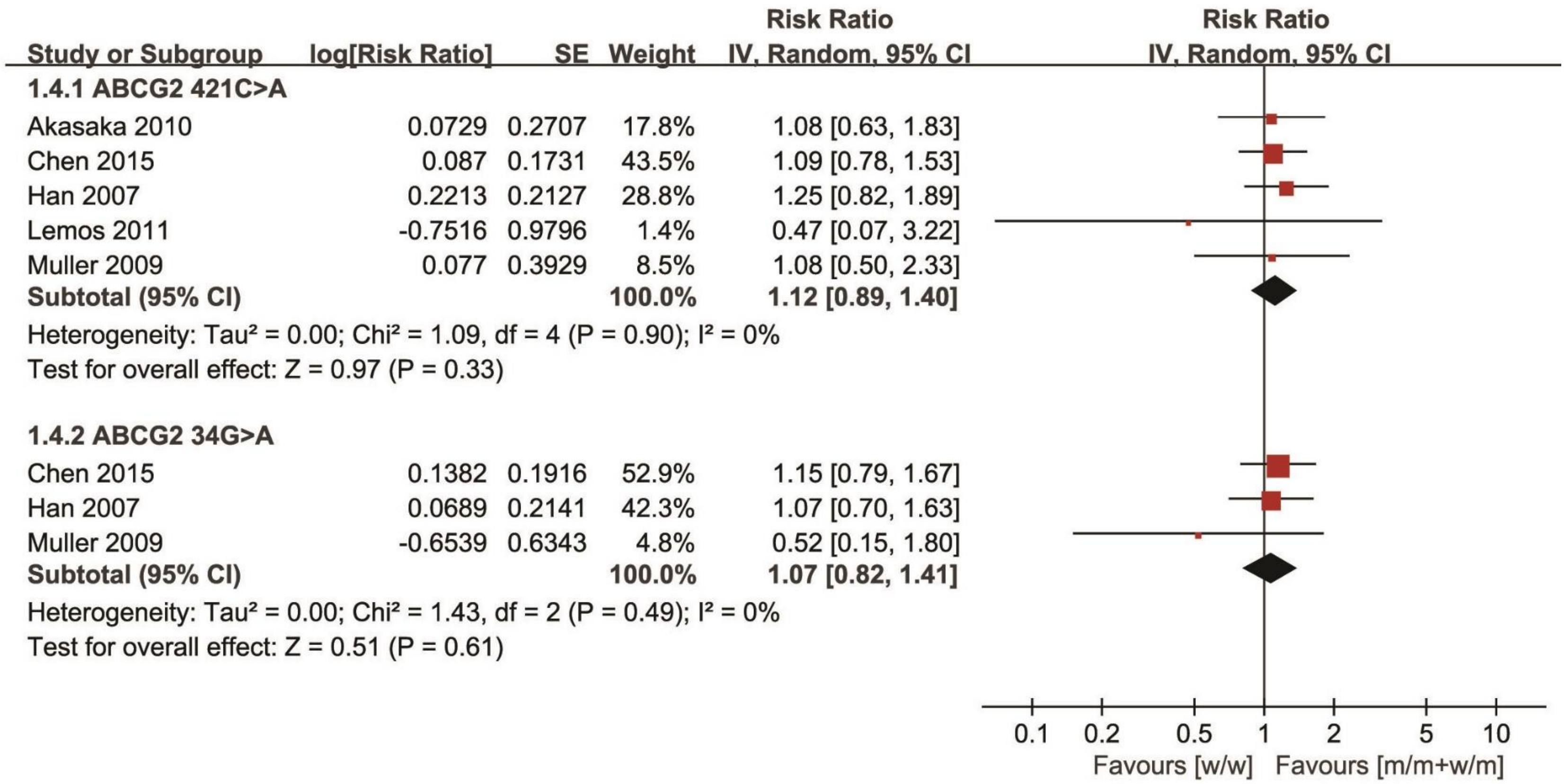

Figure S4. Meta-analysis of *ABCG2* gene polymorphism and response to chemotherapy in the NSCLC for 34 GG/(GA+AA), and 421 CC/(AC+AA).
