# Supplementary material for: A meta-analysis of ABCG2 gene polymorphism and non-small cell lung cancer outcomes
Source: Genet Mol Biol. 2020 Feb 14;42(4):e20180234. doi: 10.1590/1678-4685-GMB-2018-0234 (PMC7266279; doi:10.1590/1678-4685-GMB-2018-0234)
Supplement: Supplementary file 5 [file 1415-4757-GMB-42-4-e20180234-s5.pdf]

Supplementary Material to "A meta-analysis of *ABCG2* gene polymorphism and non-small cell lung cancer outcomes"

	m/m+w/m		w/w			Risk Ratio
Study or Subgroup	Events	Total	Events	Total	Weight	M-H, Fixed, 95% CI
**1.5.1 ABCG2 421C>A**						
Akasaka 2010	11	40	11	35	13.9%	0.88 [0.43, 1.77]
Cusatis 2006	16	24	108	143	36.8%	0.88 [0.66, 1.19]
Fukudo 2013	14	42	6	44	7.0%	2.44 [1.04, 5.76]
Han 2007	4	46	6	59	6.2%	0.86 [0.26, 2.85]
Kobayashi 2014	6	15	9	16	10.3%	0.71 [0.33, 1.51]
Lemos 2011	2	9	31	76	7.8%	0.54 [0.16, 1.91]
Ma 2017	11	27	11	21	14.7%	0.78 [0.42, 1.43]
Tamura 2012	1	38	3	45	3.3%	0.39 [0.04, 3.64]
**Subtotal (95% CI)**		**241**		**439**	**100.0%**	**0.91 [0.72, 1.15]**
Total events	65		185			
Heterogeneity: Chi² = 7.02, df = 7 (P = 0.43); I² = 0%						
Test for overall effect: Z = 0.76 (P = 0.45)						
**1.5.2 ABCG2 34G>A**						
Han 2007	6	46	5	60	26.8%	1.57 [0.51, 4.81]
Ma 2017	13	28	9	21	63.6%	1.08 [0.57, 2.04]
Tamura 2012	2	32	2	51	9.5%	1.59 [0.24, 10.76]
**Subtotal (95% CI)**		**106**		**132**	**100.0%**	**1.26 [0.73, 2.17]**
Total events	21		16			
Heterogeneity: Chi² = 0.42, df = 2 (P = 0.81); I² = 0%						
Test for overall effect: Z = 0.84 (P = 0.40)						

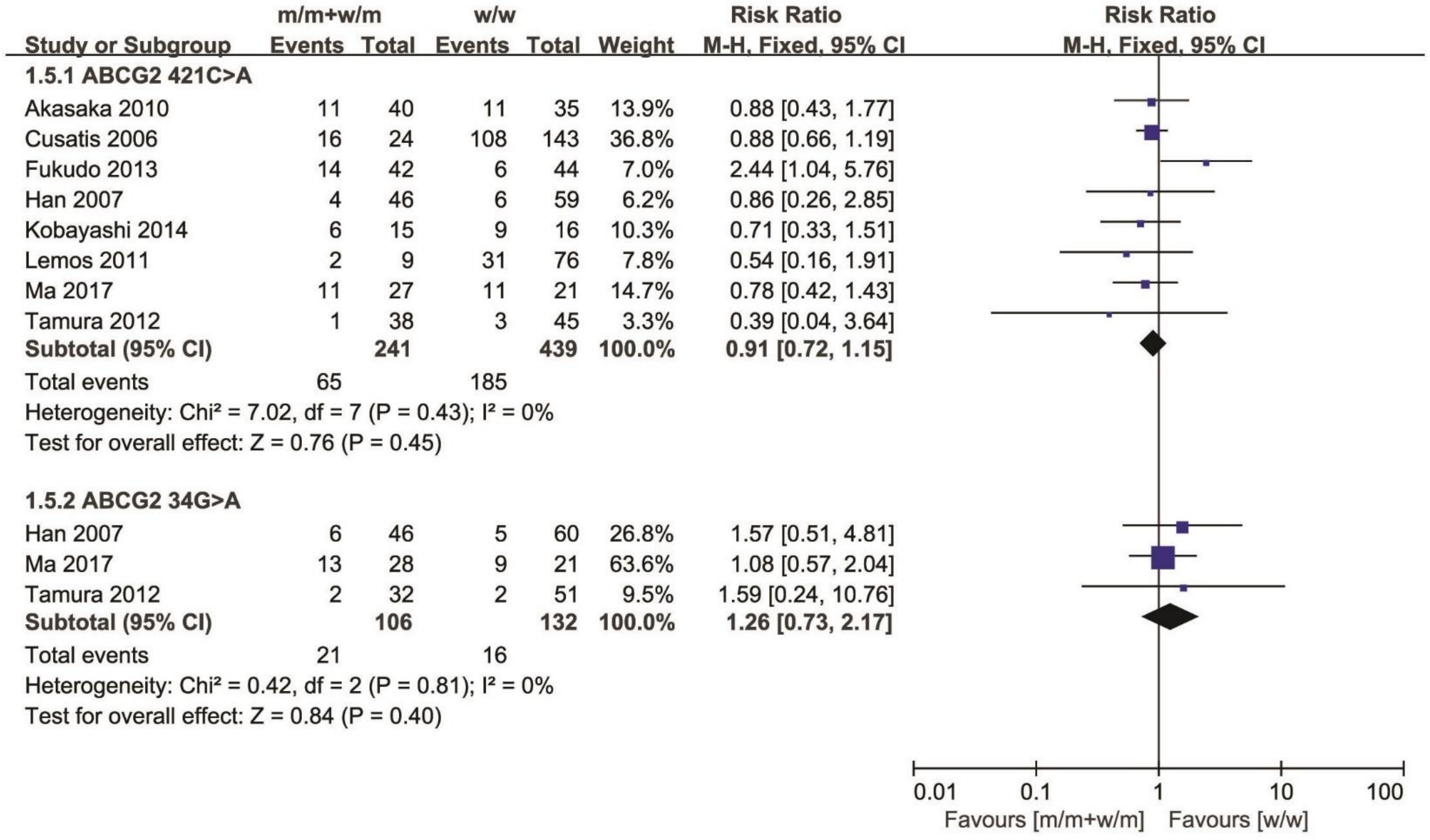

Figure S5. Meta-analysis of *ABCG2* gene polymorphism and diarrhea due to chemotherapy in the NSCLC for 34 GG/(GA+AA), and 421 CC/(AC+AA).
