# Supplementary material for: A meta-analysis of ABCG2 gene polymorphism and non-small cell lung cancer outcomes
Source: Genet Mol Biol. 2020 Feb 14;42(4):e20180234. doi: 10.1590/1678-4685-GMB-2018-0234 (PMC7266279; doi:10.1590/1678-4685-GMB-2018-0234)
Supplement: Supplementary file 6 [file 1415-4757-GMB-42-4-e20180234-s6.pdf]

Supplementary Material to "A meta-analysis of *ABCG2* gene polymorphism and non-small cell lung cancer outcomes"

	m/m+w/m		w/w			Risk Ratio
**Study or Subgroup**	**Events**	**Total**	**Events**	**Total**	**Weight**	**M-H, Fixed, 95% CI**
**1.6.1 ABCG2 421C>A**						
Akasaka 2010	29	40	27	35	21.2%	0.94 [0.72, 1.22]
Cusatis 2006	19	24	109	143	23.1%	1.04 [0.83, 1.30]
Fukudo 2013	23	42	24	44	17.3%	1.00 [0.68, 1.48]
Kobayashi 2014	8	15	12	16	8.6%	0.71 [0.41, 1.23]
Lemos 2011	4	9	42	78	6.4%	0.83 [0.39, 1.76]
Ma 2017	18	27	17	21	14.1%	0.82 [0.59, 1.15]
Tamura 2012	9	38	14	45	9.4%	0.76 [0.37, 1.56]
**Subtotal (95% CI)**		**195**		**382**	**100.0%**	**0.91 [0.79, 1.06]**
Total events	110		245			
Heterogeneity: Chi² = 3.00, df = 6 (P = 0.81); I² = 0%						
Test for overall effect: Z = 1.23 (P = 0.22)						
**1.6.2 ABCG2 34G>A**						
Ma 2017	22	28	13	21	65.8%	1.27 [0.86, 1.87]
Tamura 2012	13	32	10	51	34.2%	2.07 [1.03, 4.16]
**Subtotal (95% CI)**		**60**		**72**	**100.0%**	**1.54 [1.08, 2.21]**
Total events	35		23			
Heterogeneity: Chi² = 1.67, df = 1 (P = 0.20); I² = 40%						
Test for overall effect: Z = 2.37 (P = 0.02)						

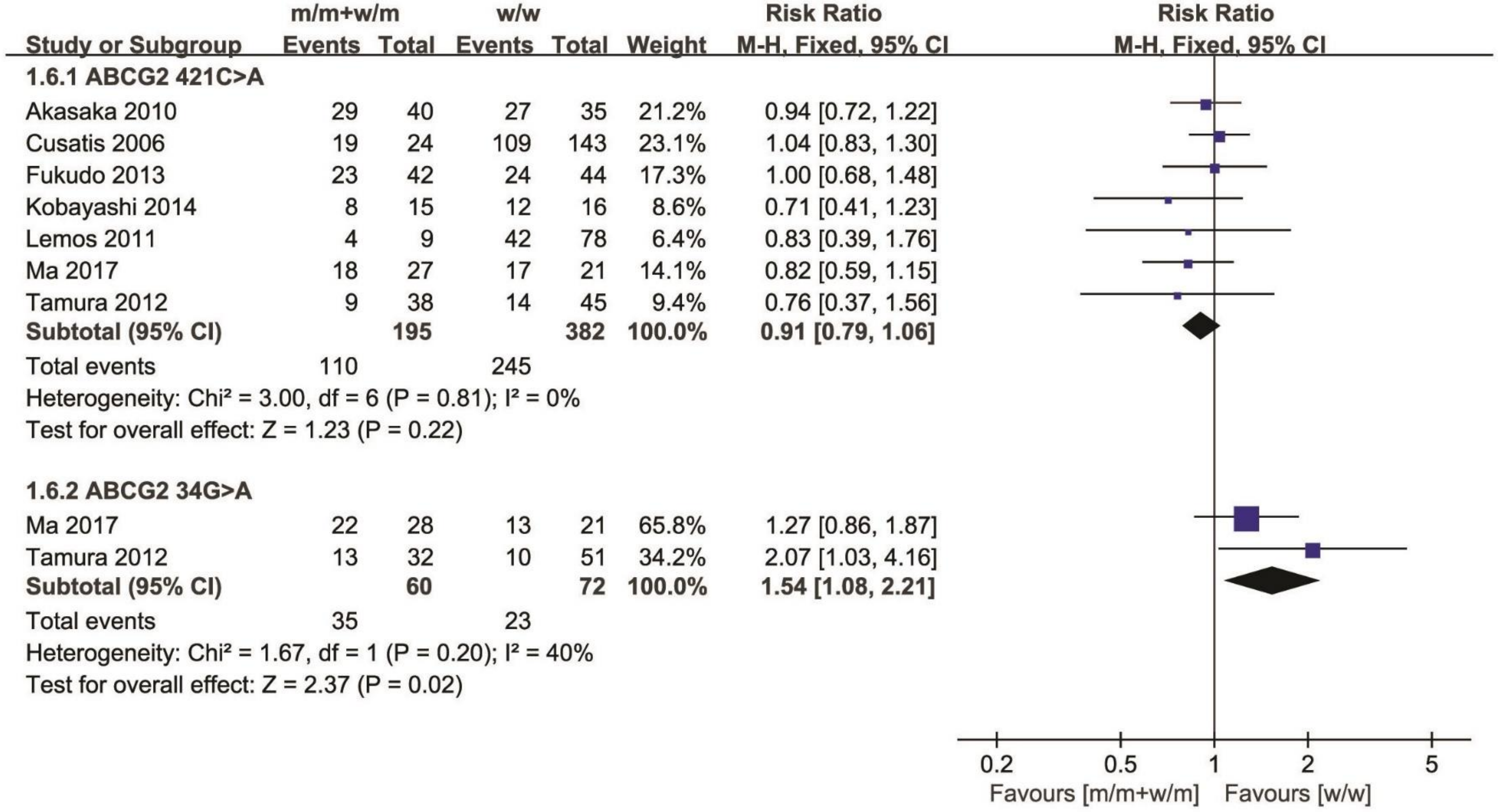

Figure S6. Meta-analysis of *ABCG2* gene polymorphism and skin toxicity due to chemotherapy in the NSCLC for 34 GG/(GA+AA), and 421 CC/(AC+AA).
