# Supplementary material for: A meta-analysis of ABCG2 gene polymorphism and non-small cell lung cancer outcomes
Source: Genet Mol Biol. 2020 Feb 14;42(4):e20180234. doi: 10.1590/1678-4685-GMB-2018-0234 (PMC7266279; doi:10.1590/1678-4685-GMB-2018-0234)
Supplement: Supplementary file 7 [file 1415-4757-GMB-42-4-e20180234-s7.pdf]

Supplementary Material to "A meta-analysis of *ABCG2* gene polymorphism and non-small cell lung cancer outcomes"

	m/m+w/m		w/w			Risk Ratio
Study or Subgroup	Events	Total	Events	Total	Weight	M-H, Fixed, 95% CI
**1.7.1 ABCG2 421C>A**						
Akasaka 2010	8	40	5	35	23.5%	1.40 [0.50, 3.89]
Kobayashi 2014	9	15	8	16	34.1%	1.20 [0.63, 2.28]
Ma 2017	8	30	2	22	10.2%	2.93 [0.69, 12.49]
Tamura 2012	7	38	8	45	32.3%	1.04 [0.41, 2.59]
**Subtotal (95% CI)**		**123**		**118**	**100.0%**	**1.37 [0.86, 2.17]**
Total events	32		23			
Heterogeneity: Chi² = 1.58, df = 3 (P = 0.66); I² = 0%						
Test for overall effect: Z = 1.34 (P = 0.18)						
**1.7.2 ABCG2 34G>A**						
Ma 2017	8	29	3	24	29.9%	2.21 [0.66, 7.41]
Tamura 2012	5	32	10	51	70.1%	0.80 [0.30, 2.12]
**Subtotal (95% CI)**		**61**		**75**	**100.0%**	**1.22 [0.58, 2.55]**
Total events	13		13			
Heterogeneity: Chi² = 1.65, df = 1 (P = 0.20); I² = 39%						
Test for overall effect: Z = 0.52 (P = 0.60)						

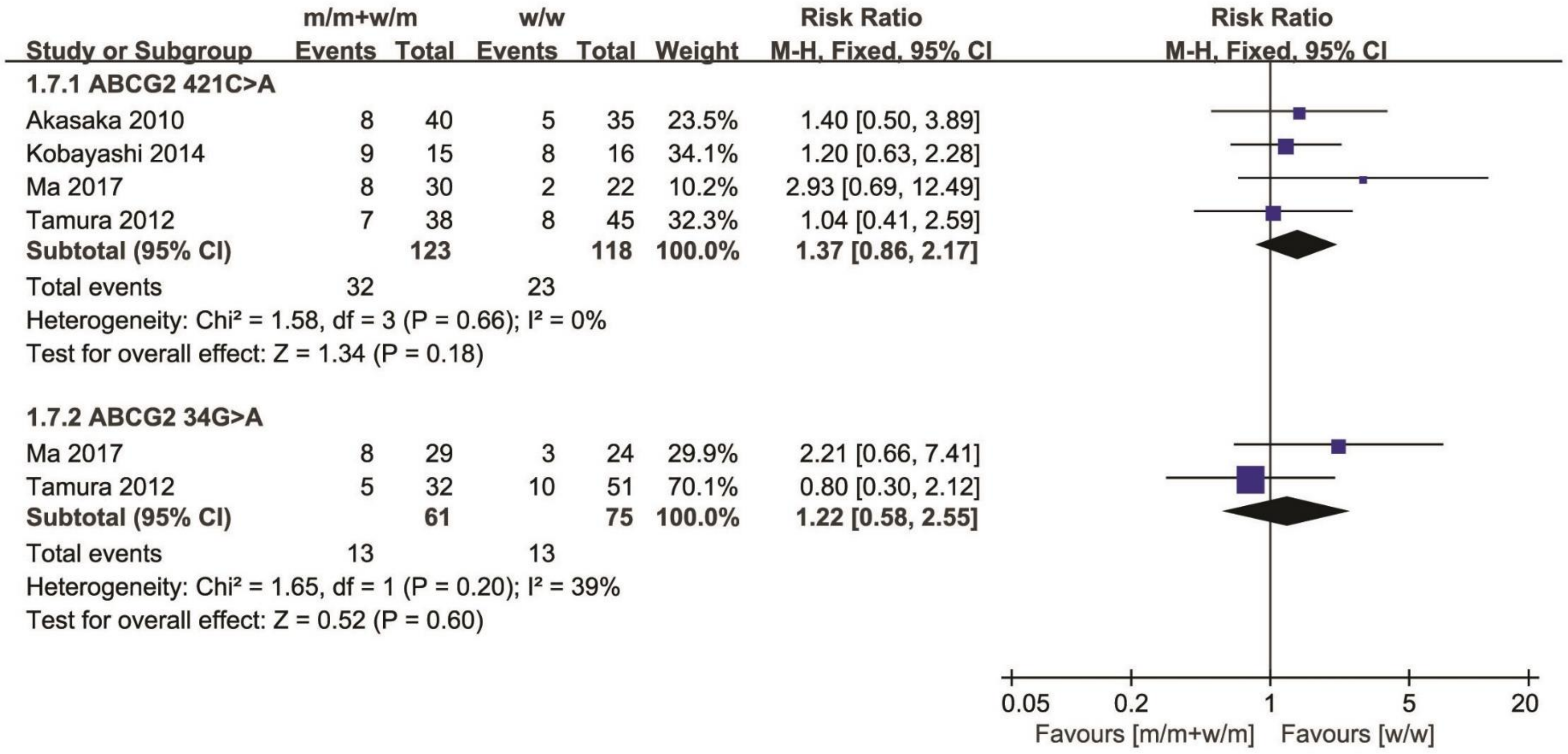

Figure S7. Meta-analysis of *ABCG2* gene polymorphism and liver dysfunction due to chemotherapy in the NSCLC for 34 GG/(GA+AA), and 421 CC/(AC+AA).
