# Supplementary material for: A meta-analysis of ABCG2 gene polymorphism and non-small cell lung cancer outcomes
Source: Genet Mol Biol. 2020 Feb 14;42(4):e20180234. doi: 10.1590/1678-4685-GMB-2018-0234 (PMC7266279; doi:10.1590/1678-4685-GMB-2018-0234)
Supplement: Supplementary file 8 [file 1415-4757-GMB-42-4-e20180234-s8.pdf]

Supplementary Material to "A meta-analysis of *ABCG2* gene polymorphism and non-small cell lung cancer outcomes"

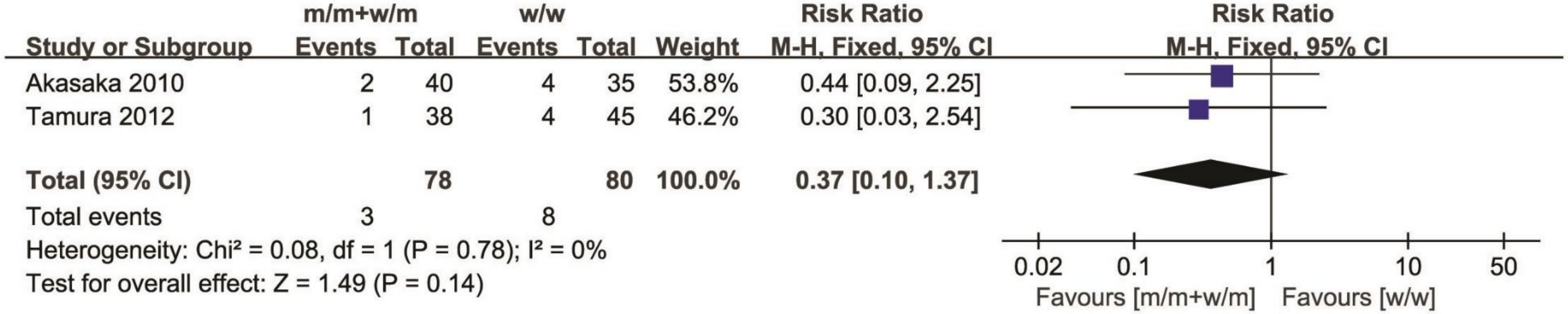

Figure S8 - Meta-analysis of *ABCG2* gene polymorphism and interstitial pneumonia due to chemotherapy in the NSCLC for 421 CC/(AC+AA).
